# Identification of potentially functional circular RNAs hsa_circ_0070934 and hsa_circ_0004315 as prognostic factors of hepatocellular carcinoma by integrated bioinformatics analysis

**DOI:** 10.1038/s41598-022-08867-w

**Published:** 2022-03-23

**Authors:** Pejman Morovat, Saman Morovat, Arash M. Ashrafi, Shahram Teimourian

**Affiliations:** 1 Department of Medical Biotechnology, Faculty of Medicine, Babol University of Medical Sciences, Babol, Iran; 2 Cellular and Molecular Biology Research Center, Health Research Institute, Babol University of Medical Science, Babol, Iran; 3 Department of Medical Genetics and Molecular Biology, School of Medicine, Iran University of Medical Sciences (IUMS), Tehran, Iran; 4 Neuroscience Research Center, Health Research Institute, Babol University of Medical Sciences, Babol, Iran

**Keywords:** Bioinformatics, Gastrointestinal cancer, Tumour biomarkers, Biological techniques, Genetics, Molecular biology, Biomarkers, Oncology

## Abstract

Hepatocellular carcinoma (HCC) is one of the most prevalent cancers worldwide, which has a high mortality rate and poor treatment outcomes with yet unknown molecular basis. It seems that gene expression plays a pivotal role in the pathogenesis of the disease. Circular RNAs (circRNAs) can interact with microRNAs (miRNAs) to regulate gene expression in various malignancies by acting as competitive endogenous RNAs (ceRNAs). However, the potential pathogenesis roles of the ceRNA network among circRNA/miRNA/mRNA in HCC are unclear. In this study, first, the HCC circRNA expression data were obtained from three Gene Expression Omnibus microarray datasets (GSE164803, GSE94508, GSE97332), and the differentially expressed circRNAs (DECs) were identified using R limma package. Also, the liver hepatocellular carcinoma (LIHC) miRNA and mRNA sequence data were retrieved from TCGA and differentially expressed miRNAs (DEMIs) and mRNAs (DEGs) were determined using the R DESeq2 package. Second, CSCD website was used to uncover the binding sites of miRNAs on DECs. The DECs' potential target miRNAs were revealed by conducting an intersection between predicted miRNAs from CSCD and downregulated DEMIs. Third, candidate genes were uncovered by intersecting targeted genes predicted by miRWalk and targetscan online tools with upregulated DEGs. The ceRNA network was then built using the Cytoscape software. The functional enrichment and the overall survival time of these potential targeted genes were analyzed, and a PPI network was constructed in the STRING database. Network visualization was performed by Cytoscape, and ten hub genes were detected using the CytoHubba plugin tool. Four DECs (hsa_circ_0000520, hsa_circ_0008616, hsa_circ_0070934, hsa_circ_0004315) were obtained and six miRNAs (hsa-miR-542-5p, hsa-miR-326, hsa-miR-511-5p, hsa-miR-195-5p, hsa-miR-214-3p, and hsa-miR-424-5p) which are regulated by the above DECs were identified. Then 543 overlapped genes regulated by six miRNAs mentioned above were predicted. Functional enrichment analysis showed that these genes are mostly associated with regulatory pathways in cancer. Ten hub genes (TTK, AURKB, KIF20A, KIF23, CEP55, CDC6, DTL, NCAPG, CENPF, PLK4) have been screened from the PPI network of the 204 survival-related genes. KIF20A, NCAPG, TTK, PLK4, and CDC6 were selected for the highest significance p-values. At the end, a circRNA-miRNA-mRNA regulatory axis was established for five final selected hub genes. This study implies the potential pathogenesis of the obtained network and proposes that the two DECs (has_circ_0070934 and has_circ_0004315) may be important prognostic markers for HCC.

## Introduction

Hepatocellular cancer (HCC) is the digestive system's most common malignancy, accounting for 75–85% of early-stage liver cancers. Its high mortality rate has become one of the main reasons for cancer-related deaths worldwide^1^. Despite advancements in treatment strategies for this type of cancer, the diagnosis of HCC patients is unsatisfactory and typically occurs in the advanced stages. Furthermore, due to the high prevalence of metastasis and recurrence after surgery as well as resistance to chemotherapy and radiotherapy, much of the most recent researches focused on improving the prognosis of HCC patients^2,3^. Therefore, there is an urgent need to understand better the molecular mechanism regulating HCC development. Leonardo Salmena et al. proposed the "competing endogenous RNA" (ceRNA) hypothesis in 2011. According to this hypothesis, ceRNAs are partial transcription products that create a vast regulatory network throughout transcriptome, including miRNA, long non-coding RNA, pseudogenic RNA, and circular RNA. These transcripts modulate each other by competing for miRNAs at the post-transcriptional level and contribute a major part to pathological situations such as cancer^4,5^. Circular RNA (circRNA) is a particular kind of endogenous non-coding RNA derived from the exonic and intronic region of a gene with a continuous and covalently closed circular structure that lacks the 5'-cap and 3'poly-A tail. So, it is more resistant to degradation by exonucleases and RNases, giving it a better stability than linear RNAs. CircRNAs are physiologic and tissue-specific, making them ideal diagnostic and potential prognostic biomarkers in tissues, serum, and urine^6–8^.

CircRNAs participate in several biological processes, including transcriptional and post-transcriptional regulation. These transcripts contain miRNA response element (MRE) sites, making them function as ceRNAs, limiting miRNA function through complementary base pairing and indirectly regulating expression of their downstream target genes via interactions with RNA binding protein and modulating mRNA stability. Some studies have shown that circRNAs, which function as ceRNAs, contribute to malignancies such as bladder cancer, hypopharyngeal carcinoma, and breast cancer. Therefore, it seems they may have an excellent potential use in the therapeutic approaches^9–11^.

Emerging evidence has discovered that circRNAs are differentially expressed in liver cancer cells and act as oncogenes in HCC^12,13^. For instance, Xiufeng Yu et al. demonstrated that circMAST1 circular RNA is upregulated in HCC tissues, leading to cell proliferation and migration, which sponges miR-1229 and regulates CTNND1 expression^14^.

**Figure 1:**
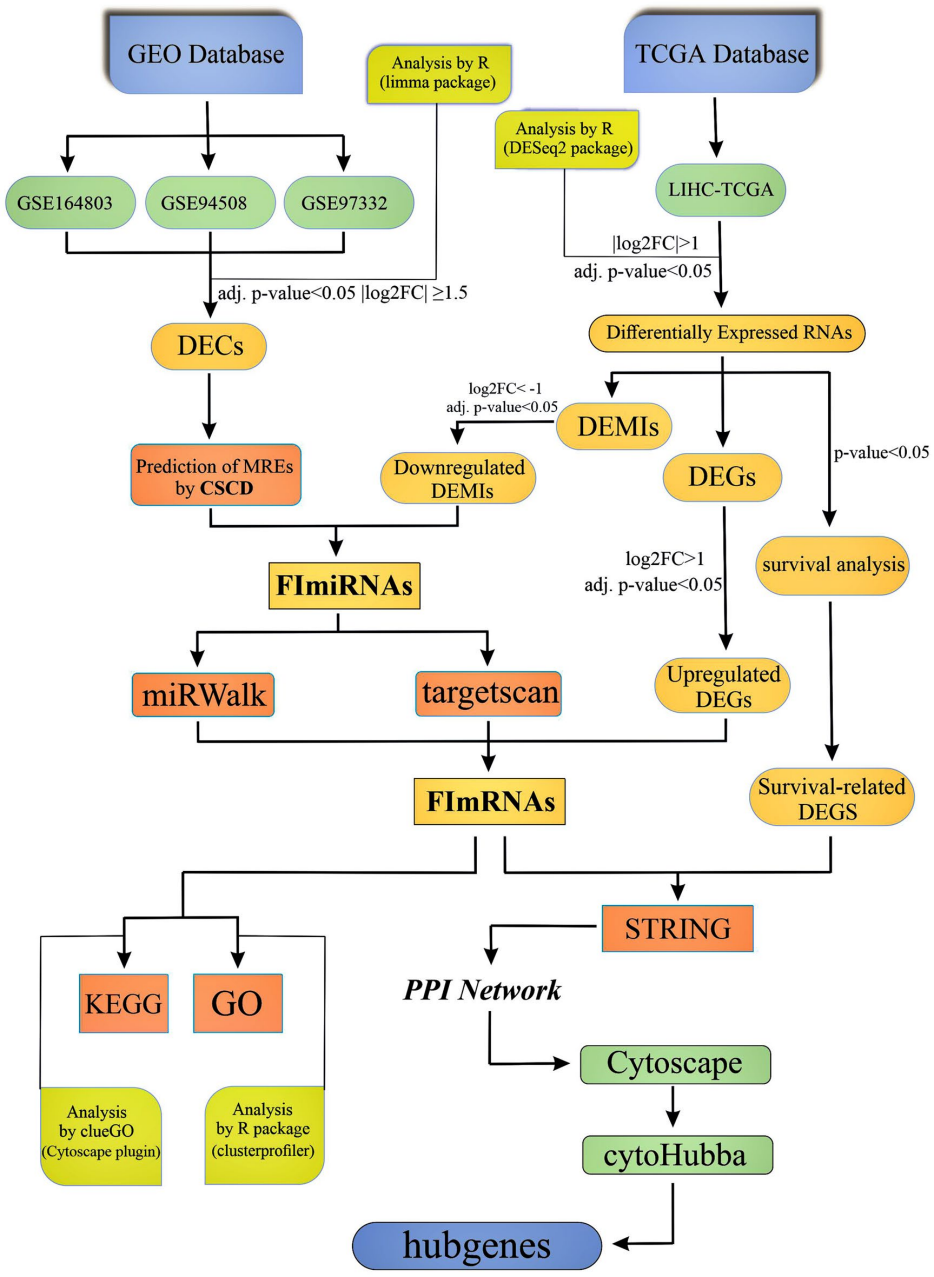
Flowchart of this study.

However, other studies suggest that circRNAs may act as tumor suppressors in hepatocellular carcinoma cells^15,16^. For example, Dan Han et al. showed that circMTO1 sponges oncogenic microRNA-9 and leads to upregulation of p21 expression, which inhibits HCC development^17^. These findings suggest that circRNAs should be explored further as potential biomarkers and therapeutic targets in HCC patients. Many circRNAs have already been discovered due to recently improved high-throughput RNA sequencing technologies; nevertheless, their potential mechanism in HCC is still not fully understood. In this research, bioinformatic analysis was used to investigate the possible methods by which particular novel circRNAs may function as ceRNAs to sponge miRNA and regulate mRNA expression in HCC. The flowchart of this study is depicted in Fig. 1**.**

The National Center for Biotechnology Information Gene Expression Omnibus (NCBI GEO)^18^ was used to acquire microarray expression profiles of HCC-related circRNAs, and The Cancer Genome Atlas (TCGA)^19^ was used to retrieve miRNA and mRNA sequencing data. Then, using R packages, an integrated analysis was conducted to detect differentially expressed circRNAs (DECs), miRNAs (DEMIs), and mRNAs (DEGs). Additionally, online tools and screening were employed to obtain DECs-related miRNAs and miRNA target genes to construct a circRNA/miRNA/mRNA regulatory network. Functional enrichment and pathway enrichment were performed on all mRNAs in the network to discover additional biological activities among these molecules. Furthermore, a protein–protein interaction (PPI) network for survival-related mRNAs was successfully built, and hub genes were detected.

## Materials and methods

### Microarray data collection and identification of differentially expressed circRNAs (DECs)

To identify differentially expressed circRNAs (DECs), three microarray datasets containing circRNA expression profiles in HCC patients were downloaded from GEO (https://www.ncbi.nlm.nih.gov/geo/) as a functional genomic database by using GEOquery package in R language software (V 4.1.0). The gene chips in all eligible microarray datasets have the same platform (Agilent-069978 Arraystar Human CircRNA microarray V1). The microarray data consist of three datasets:

GSE164803 (six normal tissue samples and six HCC tissue samples), GSE94508 (five paracancerous tissue samples and five HCC tissue samples), and GSE97332 (seven hepar normal tissue samples and seven HCC tissue samples), Additionally, all raw expression data were normalized and log_2_transformed. The sva package (V 3.40.0)^20^in R software was also used to remove the hidden batch effects and combine three datasets. The R limma package (V 3.48.3)^21^ based on the Bioconductor package was applied to screen DECs microarray datasets with the cutoff criteria of |log_2_FoldChange|≥ 1.5 and adj. p-value < 0.05. This criterion was considered statistically significant.

### Identification of differentially expressed miRNAs (DEMIs) and differentially expressed mRNAs (DEGs)

On July 29, 2021, the RNA-Seq and miRNA-Seq expression data of hepatocellular liver carcinoma (LIHC) were retrieved from The Cancer Genome Atlas (TCGA), a public database that indicates genomic alterations related to cancer. There are 371 primary tumors and 50 normal solid tissue for RNAs, while there are 372 primary tumors and 50 normal solid tissue for miRNAs. Clinical data on liver cancer were extracted through TCGA's GDC portal. (https://portal.gdc.cancer.gov/).

After removing duplicate samples and other tissue samples with incomplete clinical stages and vital status information, the final number of RNA samples in primary tumor and normal solid tissue decreased to 347 and 42, respectively, and the final number of miRNA samples in primary tumor and normal solid tissue decreased to 348 and 42, respectively.

RNA-Seq and miRNA-Seq data were normalized by the R package GDCRNATools^22^ utilizing TMM^23^ and VOOM^24^ methods. Expression data analysis for RNA and miRNA between primary tumor and normal solid tissue was performed by DESeq2 (V 1.32.0), a Bioconductor package based on R language to gain differentially expressed miRNAs (DEMIs) and differentially expressed mRNAs (DEGs). DESeq2 performs an internal normalization by computing the geometric mean for each gene across all samples. The number of genes in each sample is then divided by the mean value^25^. The threshold for selecting DEMIs and DEGs based on two-dimension, |log_2_FoldChange|> 1 and adj. p-value < 0.05, these two statistical thresholds were considered significant.

### Prediction of miRNA response elements (MREs)

The Cancer-Specific CircRNA Database (CSCD, http://gb.whu.edu.cn/CSCD/) is a web-based tool for understanding the functional effects of circRNAs that was used to predict miRNA Response Element (MRE) sites for each DECs. These target miRNAs, which are complementary to the DECs-related MREs, have been named CTmiRNAs (CSCD target miRNAs).

CTmiRNAs were further screened according to the DEMIs obtained from the TCGA database. To acquire potential target miRNAs on the DECs, an intersection was performed between CTmiRNAs and downregulated DEMIs (downregulated DEMIs were selected with the cutoff criteria of log_2_FoldChange < −1 and adj. p-value < 0.05). Finally, the intersection and overlapping of these two algorithms' miRNAs have been dubbed FImiRNAs (Final intersected miRNAs).

### Prediction of miRNA–mRNA interaction

The Targetscan (http://www.targetscan.org/vert_72) and Mirwalk (http://mirwalk.umm.uni-heidelberg.de) websites were used to predict target genes. These two mentioned websites are comprehensive publicly available online resources of miRNA-target interactions. Overlapped miRNA target genes between these two databases are considered final target genes, which we termed FmRNAs (Final mRNAs).

To identify potential miRNA-related genes, the intersection of upregulated DEGs from TCGA-RNA-seq analysis and FmRNAs was performed using the same method. (upregulated DEGs were selected with the cutoff criteria of log_2_FoldChange > 1 and adj. p-value < 0.05). At last, we got the final functional genes, which we refer to as FImRNAs (Final Intersected mRNAs).

### Formation of circRNA/miRNA/mRNA regulatory network

Using previous analysis and predicted target interactions, the acquired DEC-FImiRNA pairs, and FImiRNA-FImRNA pairs were combined to form a circRNA/miRNA/mRNA regulatory network, according to the ceRNA hypothesis. This network was visualized using the Cytoscape software (V 3.8.2)^26^, a robust tool for analyzing and visualizing data networks.

### Functional enrichment analysis for FImRNAs

Gene ontology (GO)^27^ and Kyoto Encyclopedia of Genes and Genomes (KEGG)^28^ analysis were applied to explore the main function and associated enriched pathways information of the FImRNAs in the preliminary ceRNA regulatory network.

Using the GO database, GO assessed for the biological process (BP), cellular component (CC), and molecular function (MF) of each element to find potential functional genes. ClusterProfiler (V 4.0.2) is an R software package based on ontology for statistical analysis and visualizing gene functional profiles^29^. In this analysis, the ClusterProfiler with the Benjamini–Hochberg method and p-value < 0.05 and q-value < 0.05 criteria were utilized for GO analysis of FImRNAs. Based on the KEGG database, KEGG determines the potential functions of these genes that participated in the pathways. ClueGO, a Cytoscape plugin, was used to perform and visualize the KEGG analysis^30^. Functional annotation with a p-value below 0.05 and a kappa score greater than 0.4 was considered statistically significant.

### Survival analysis of FImRNAs

Kaplan–Meier method was used to evaluate the overall survival of DEGs in 347 HCC samples using the survival tool package. The hazard ratio (HR) and corresponding 95% confidence interval were calculated, and a p-value less than 0.05 was considered statistically significant. Lastly, the intersection of survival-related DEGs and FImRNAs was conducted.

### Construction of protein–protein interaction (PPI) network and identification of hub genes

The STRING (https://string-db.org/), an online search tool for predicting protein–protein interactions (PPI) used to establish a PPI network^31^. The overlapped findings for the overall survival of DEGs and FImRNAs were imported into the STRING database. The extraction cutoff standard for the PPI pair was used with an interaction score greater than 0.4. The obtained network was visualized using Cytoscape, and hub genes were found using the Cytoscape plugin, CytoHubba, a network analysis tool with a simple user interface and 11 scoring systems was used to evaluate the significance of nodes in a biological network^32^.

## Results

### Identification of DECs, DEMIs, and DEGs

The differential expressed circRNAs (DECs) were verified in the initial step to establish an interaction network among the circRNA–miRNA–mRNA regulatory axis. Three microarray datasets from GEO on an identical platform from HCC patients were included in this study.

**Figure 2:**
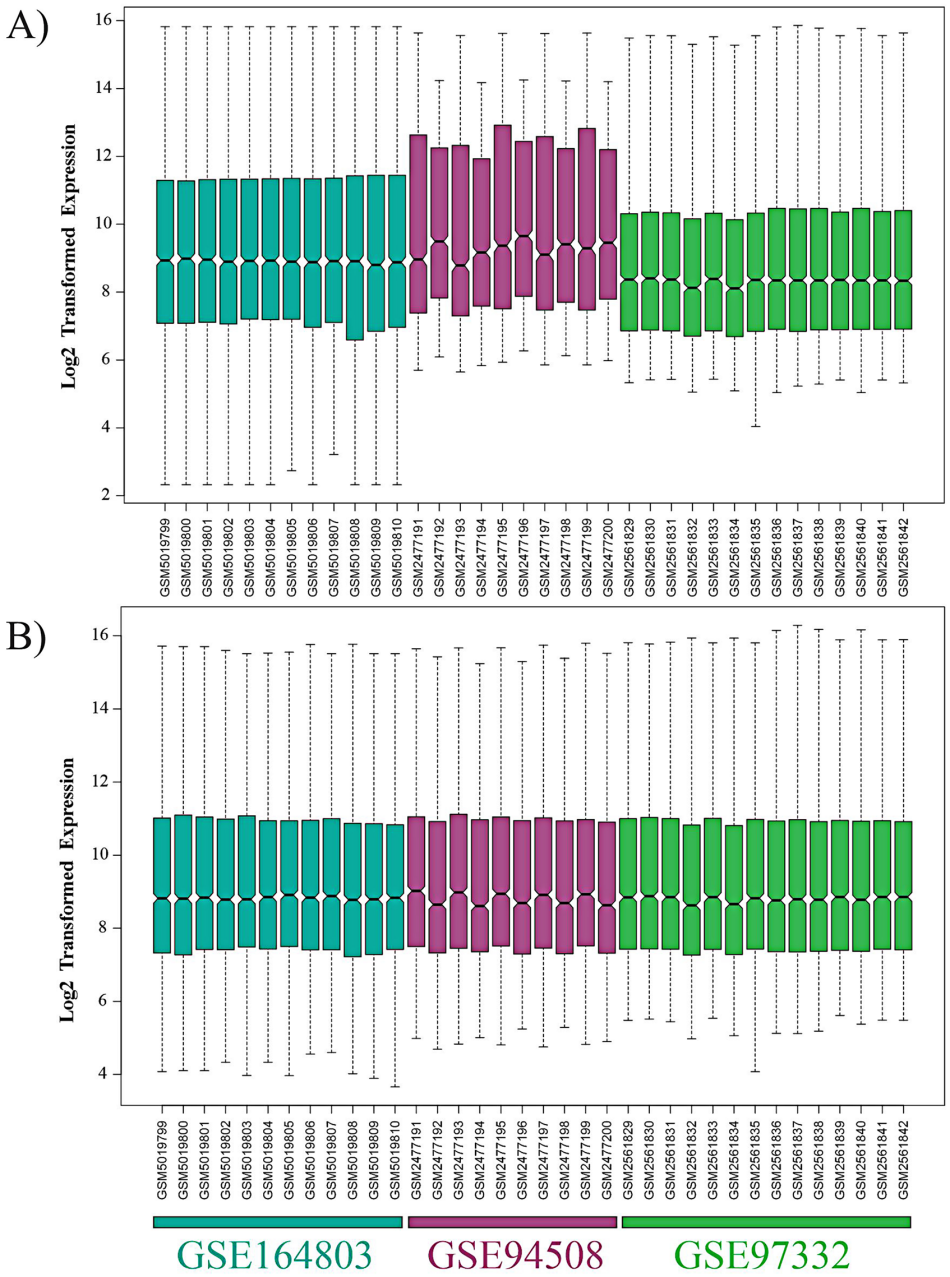
The box plots illustrate the overall expression profiles of three Affymetrix gene chips (**A)** after being normalized by normalize quantiles method, (**B)** after batch effect removal by ComBat method using the sva package in R software.

First, the raw expression data was log_2_transformed in R software and then normalized using the NormalizeQuantiles function (Fig. 2A). Following the merging of these three datasets, the popular ComBat function in the R sva package was used to eliminate the hidden batch effects (Fig. 2B). We utilized the R limma package for differential analysis of all microarray data and got a string of statistically significant circRNAs comprising 2042 variables. Nine circRNAs with much more significant differential expression than other circRNAs were identified as the main DECs including hsa_circ_0083766, hsa_circ_0074854, hsa_circ_0008616, hsa_circ_0079958, hsa_circ_0000520, hsa_circ_0004315, hsa_circ_0091570, hsa_circ_0016867, and hsa_circ_0070934 by the criteria adj. p-value < 0.05 and ǀlog_2_FoldChangeǀ > 1.5.

**Figure 3:**
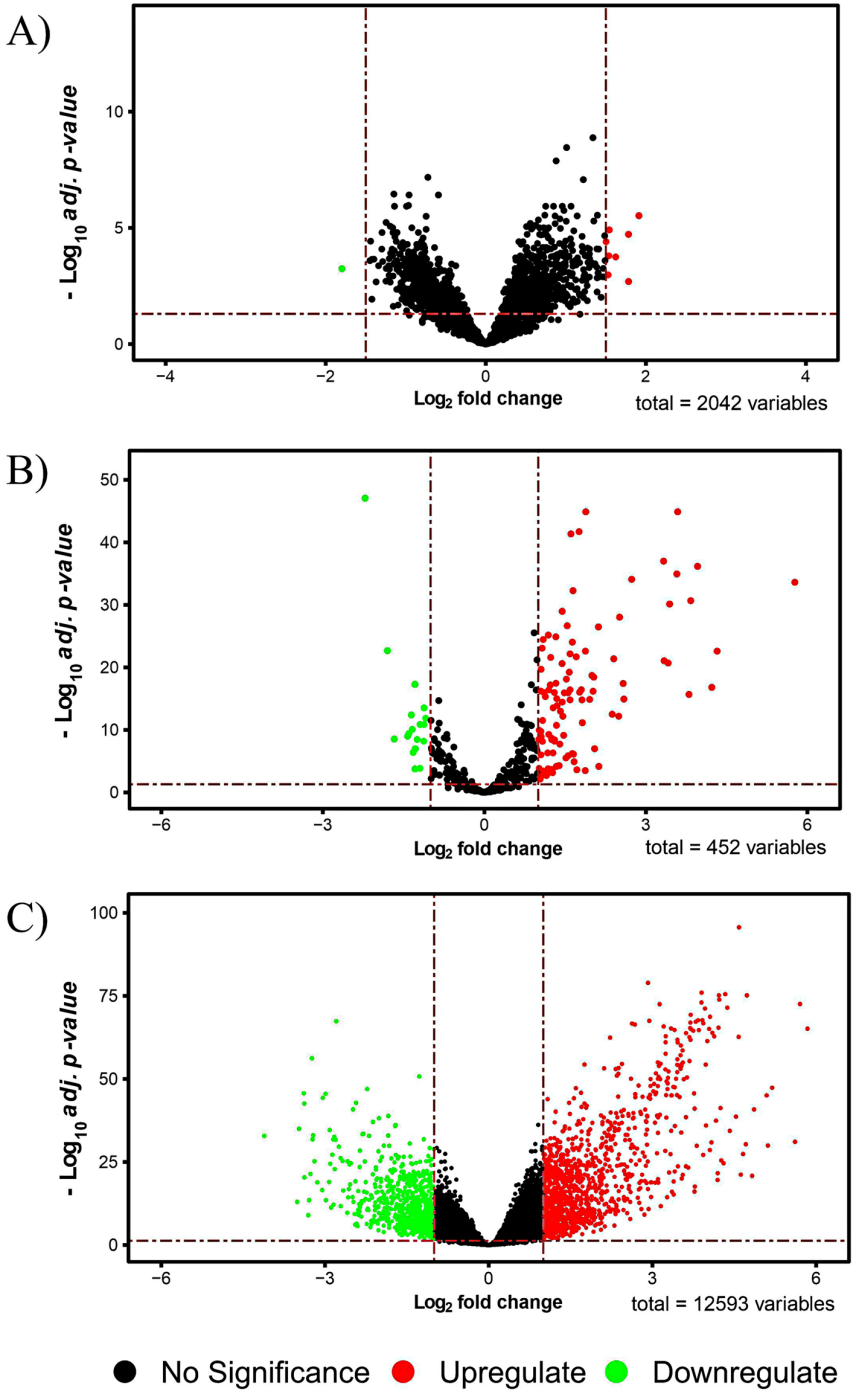
Volcano plots for (**A)** differentially expressed circRNAs (DECs), (**B)** differentially expressed miRNAs (DEMIs), (**C)** differentially expressed mRNAs (DEGs). The green and red points on the graph indicate down and up expressed, respectively. Volcano plots were generated using the R package *"*EnhancedVolcano*".*

This set of DECs in HCC tissues were found compared to normal tissues. Eight were upregulated, and one was downregulated circRNA (Fig. 3A). After further analysis and filtering among these nine DECs, four upregulated DECs, including hsa_circ_0000520, hsa_circ_0008616, hsa_circ_0070934, hsa_circ_0004315, were chosen as the research objects in this study (Table 1).

**Table 1:** Basic information of the four DECs.

Differentially expressed circRNA	Position	Gene symbol	Adj. p-value	logFC	Regulation
hsa_circ_0000520	chr14:20811436–20811559	RPPH1	1.90E−05	1.78	Up
hsa_circ_0004315	chr16:74491771–74493687	GLG1	0.001	1.53	Up
hsa_circ_0008616	chr16:19619499–19628130	C16orf62	0.0002	1.62	Up
hsa_circ_0070934	chr4:128995614–129012667	LARP1B	0.002	1.78	Up

In the second step of building a circRNA/miRNA/mRNA network, verifying differentially expressed miRNAs (DEMIs) and differentially expressed mRNAs (DEGs) was essential. DESeq2 package analysis in R was performed on samples taken from the TCGA-LIHC database; as a result, the DEMIs and DEGs between primary tumor and normal solid tissue were revealed. DEMIs consisted of 108 upregulated and 19 downregulated genes (Fig. 3B), whereas DEGs consisted of 1229 upregulated genes and 797 downregulated genes (Fig. 3C). The DEGs and DEMIs were determined using the criteria log_2_FoldChange > 1 and adj. p-value < 0.05.

### Prediction of MREs and their corresponding target miRNAs

**Figure 4:**
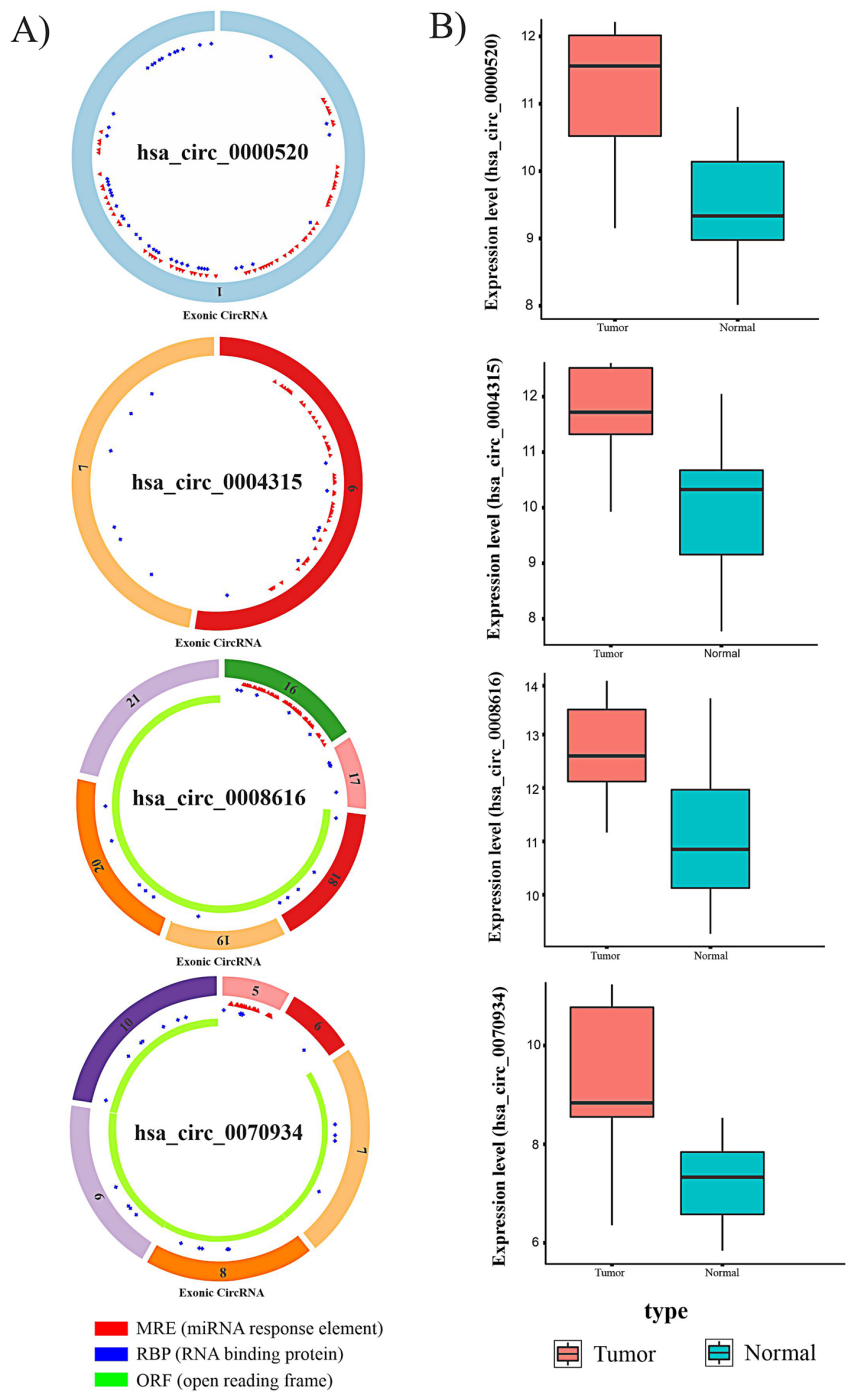
**(A)** The basic structure of four DECs with MRE, RNA binding protein, and open reading frame information. The schema graphs of four DECs information were downloaded from the CSCD website. (**B)** Transcription levels of four DECs with variable expression levels between normal and tumor samples.

Several research studies have been published on the function of circRNAs that may serve as ceRNAs in different cancers. These data suggest that circRNAs trap miRNAs like a sponge. The four DECs identified contain miRNA response element (MRE) sites that sponge miRNAs. (Fig. 4A) We used the CSCD database to predict potential target miRNAs of these DECs; we called the predicted miRNAs derived from CSCD, CTmiRNAs.

Because the DECs selected for this study were upregulated and, according to the ceRNA hypothesis, circRNAs lead to miRNA downregulation, we intersected the CTmiRNAs with 19 downregulated DEMIs from the TCGA-LIHC analysis and called them FImiRNAs (hsa-miR-542-5p, hsa-miR-326, hsa-miR-511-5p, hsa-miR-195-5p, hsa-miR-214-3p, and hsa-miR-424-5p). Table 2 displays the total number of DEC-miRNA interactions, and Fig. 4B depicts the expression levels of four DECs in normal and tumor samples.

**Table 2:** circRNA-miRNA interactions.

Differentially expressed circRNA	The MREs corresponding target miRNA (FImiRNA)
hsa_circ_0000520	hsa-miR-542-5p
hsa_circ_0008616	hsa-miR-326 hsa-miR-542-5p
hsa_circ_0070934	hsa-miR-511-5p
hsa_circ_0004315	hsa-miR-195-5p hsa-miR-214-3p hsa-miR-424-5p

### Prediction and analysis of miRNA-mRNA pairs and construction of ceRNA network

MiRNAs may bind to complementary sequences inside mRNAs, leading to their degradation or suppression; thus, the Targetscan and Mirwalk databases have been used to predict and determine which RNAs have complementary binding sites to bind our selected FImiRNAs. After that, FmRNA was chosen as an appropriate term for the final target genes earned from the intersection of two databases.

Upregulation of DECs, as stated by the ceRNA hypothesis, causes downregulation of corresponding target miRNAs. Since fewer miRNAs are in the cytoplasm, target gene transcripts are less degraded and suppressed. Therefore, we carried an intersection between FmRNAs and upregulated DEGs; as a result, 543 genes were discovered. In this study, these 543 final intersected mRNAs were termed FImRNAs.

**Figure 5:**
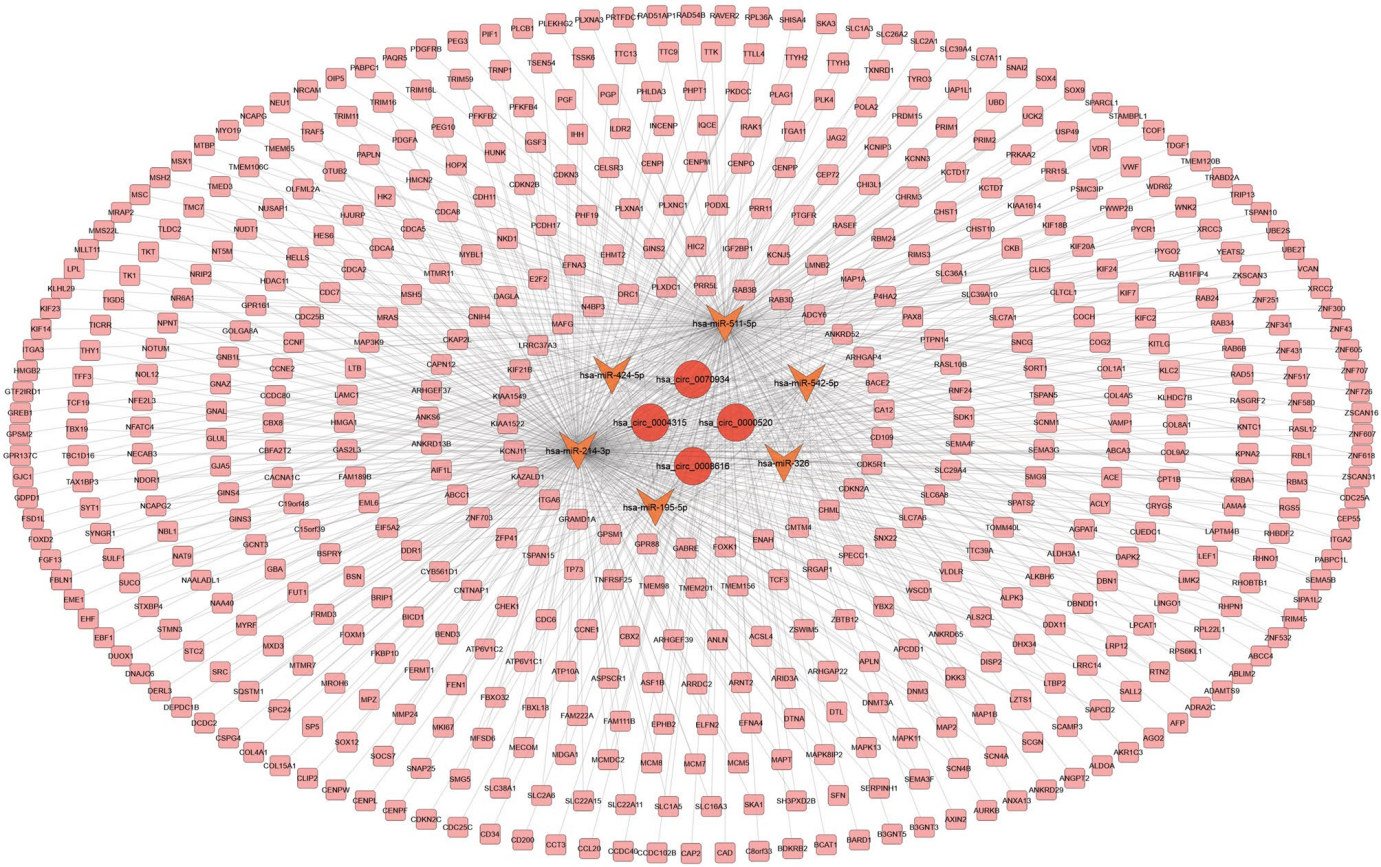
ceRNA network. The network consisting of four DECs (hsa_circ_0000520, hsa_circ_0008616, hsa_circ_0070934, hsa_circ_0004315) and six FImiRNAs (hsa-miR-542-5p, has-miR-326, has-miR-511-5p, has-miR-195-5p, has-miR-214-3p, has-miR-424-5p) and 543 FImRNAs. Ellipse represents circRNA, V represents miRNA and Round Rectangle represents mRNA.

To better understand the roles of DECs on mRNAs through miRNA binding in HCC cancer, a ceRNA network was constructed and visualized by Cytoscape software for four selected DECs, their potential target miRNAs (DEC-FImiRNA pairs), and miRNA target genes (FImiRNA-FImRNA pairs). We utilized the downregulated miRNA as a junction in this network, along with upregulated circRNAs and mRNAs in the circRNA-miRNA and miRNA-mRNA interaction pairings. Finally, there were 300 interactions in the ceRNA network, including four DECs, six FImiRNAs, and 543 FImRNAs (Fig. 5).

### GO and KEGG pathway enrichment analysis of FImRNAs

**Figure 6:**
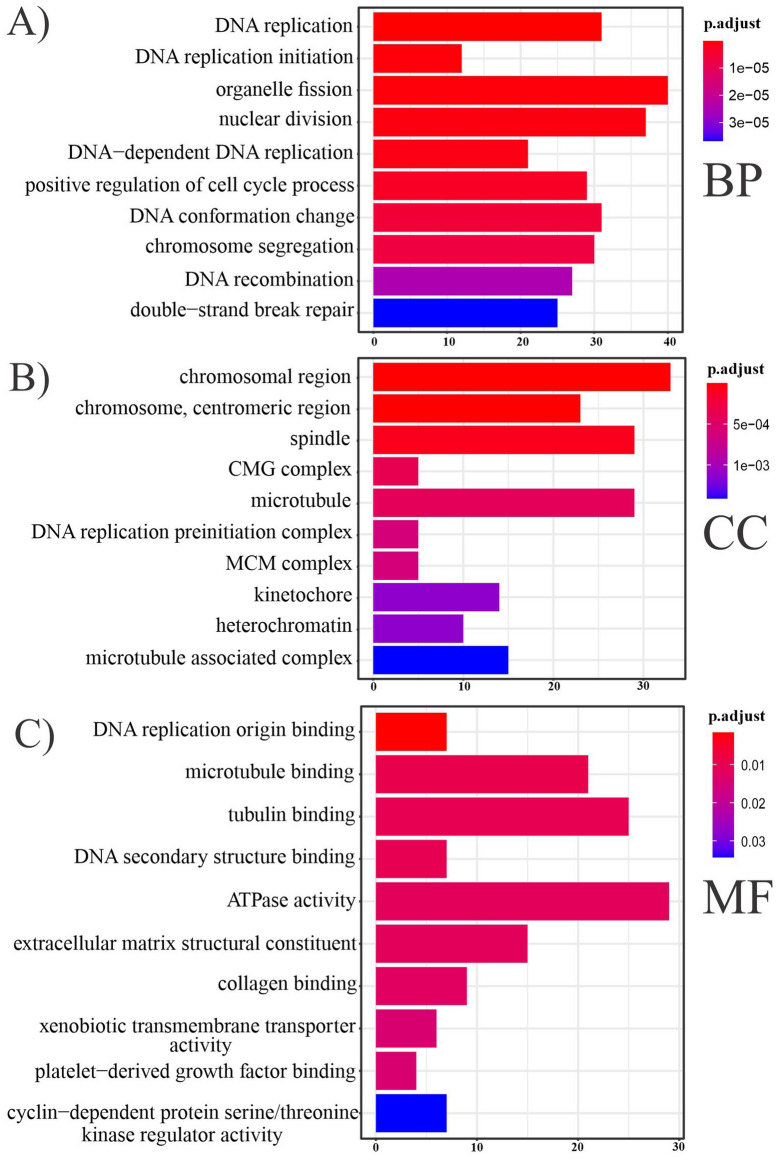
Bar plots show an overview of GO analysis, each bar plot displaying ten remarkably enriched terms in three categories (**A)** biological process, (**B)** cellular component, (**C)** molecular function. The terms at the top of the barplot are more significant than the other terms.

Using the ClusterProfiler R package, GO analysis including three BP, CC, and MF categories was used to understand better the biological roles of 543 FImRNAs (Fig. 6). 4199 BPs, 499 CCs, and 748 MFs were enriched for this dataset. P-value with a cutoff set less than 0.05 was considered statistically significant.

**Figure 7:**
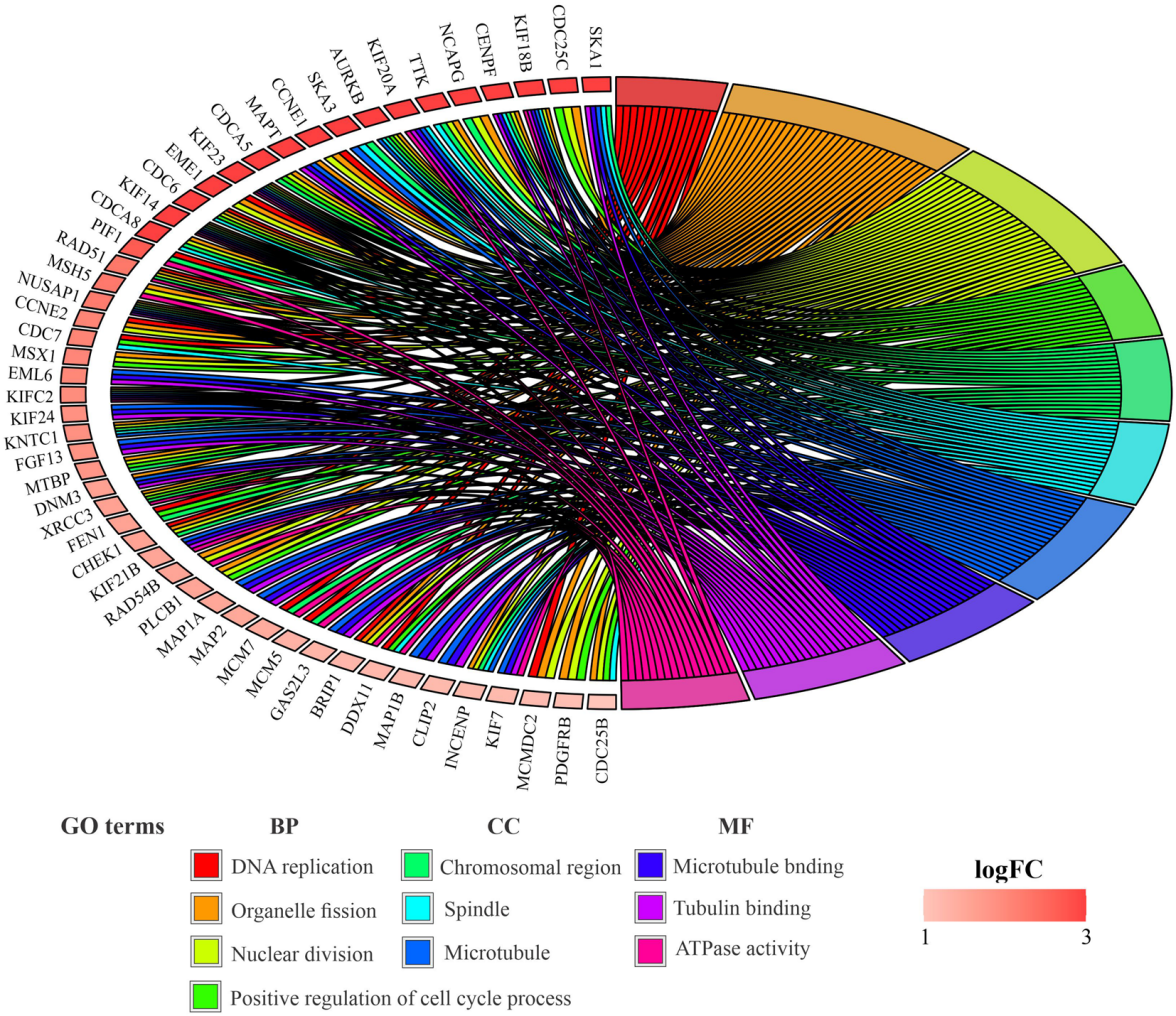
Chord plot of FImRNAs that enriched in the top four terms associated with BP, three CC terms, and three MF terms. Chord plot was generated via the R package *"*GOplot*".*

Based on the count of genes in the pathway and the degree of significance for BPs terms, these FImRNAs were mainly enriched in organelle fission, nuclear division, DNA replication, positive regulation of cell cycle process, and DNA conformation changes, respectively. Likewise, for MF terms, according to the number of genes in the pathway and the degree of significance, these FImRNAs are mainly enriched in ATPase activity, tubulin binding, microtubule-binding, extracellular matrix structural constituent, DNA replication origin binding, respectively. For CC terms, these FImRNAs are mainly enriched in the chromosomal region, spindle, microtubule, chromosome, centromeric region, and kinetochore, respectively, depending on the count of genes included in the pathway and the degree of significance. The chord plot indicates that 51 of 543 FImRNAs had high log_2_FoldChange values and were enriched in the top four BP terms, three CC terms, and three MF terms (Fig. 7).

**Figure 8:**
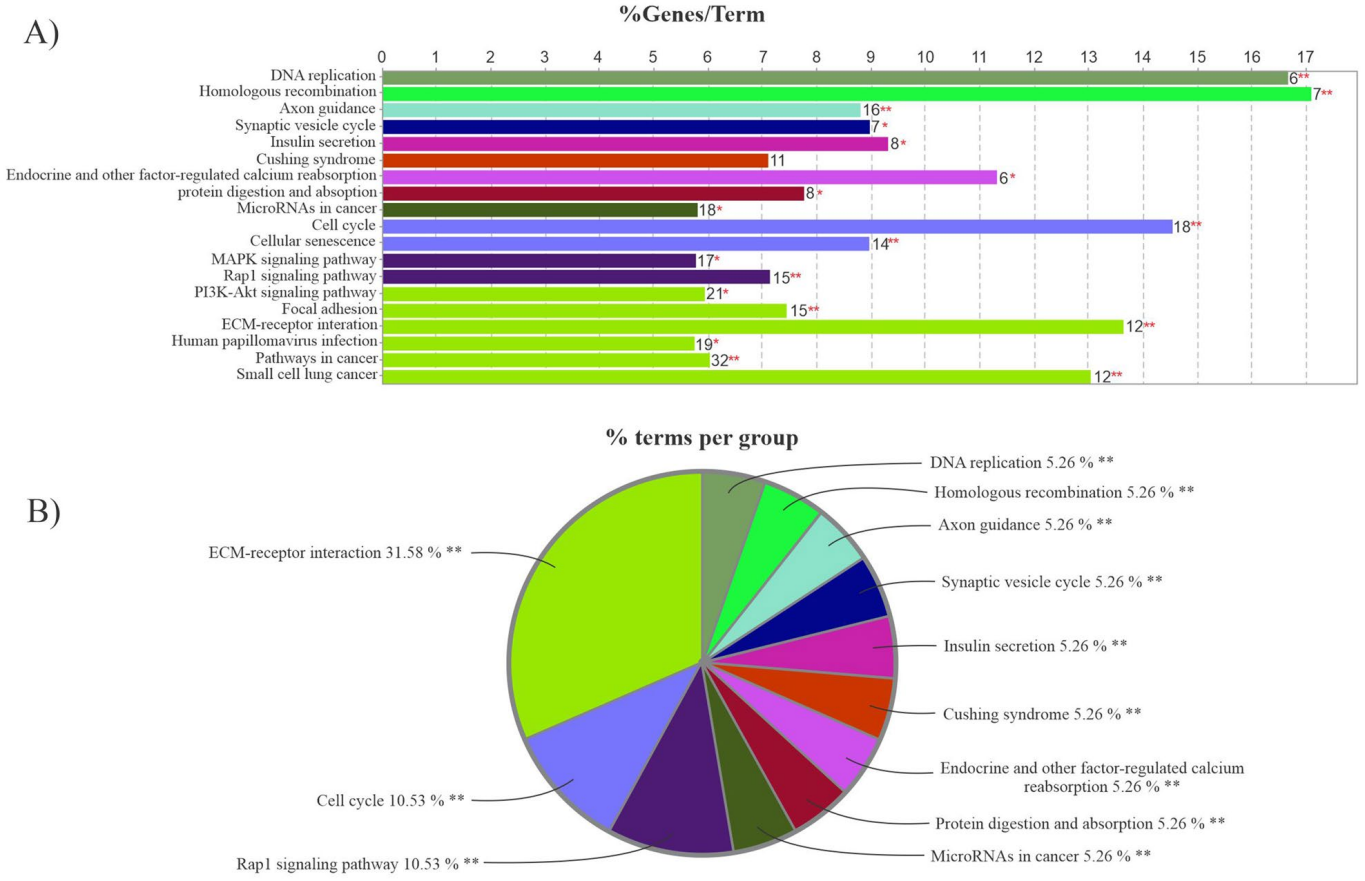
KEGG pathway analysis. (**A)** The bars in the chart represent the number of analyzed genes associated with each term. The numbers at the top of the bars indicate the percentage of detected genes relative to all of the genes related to the term. (**B)** The pie chart depicts an overview of the functional groups for FImRNAs. Groups are named based on the most significant term in each group. The bar plot and pie chart were visualized using clueGO (Cytoscape plugin).

KEGG analysis was done on 543 FImRNAs using clueGO, a Cytoscape plugin, to reveal their potential function (Fig. 8). The statistical analysis criteria were p-value ≤ 0.05 and a kappa score ≥ 0.4. According to the charts, the groups' network between terms, the KEGG signal pathway enrichment analysis suggests that these FImRNAs were enriched in pathways such as ECM-receptor interaction, cell cycle, and Rap1 signaling pathway.

### Survival analysis and construction of PPI network

The Kaplan–Meier survival analysis was conducted to analyze the connection between DEGs and survival time. As an outcome, 441 DEGs with p-value survival < 0.05 were identified between primary tumor and normal solid tissue. In order to evaluate the survival analysis of the ceRNA network established in the previous results, an intersection was accomplished between 441 survival-related DEGs and 543 FImRNAs, resulting in the identification of 204 genes associated with the overall survival of the HCC patients.

**Figure 9:**
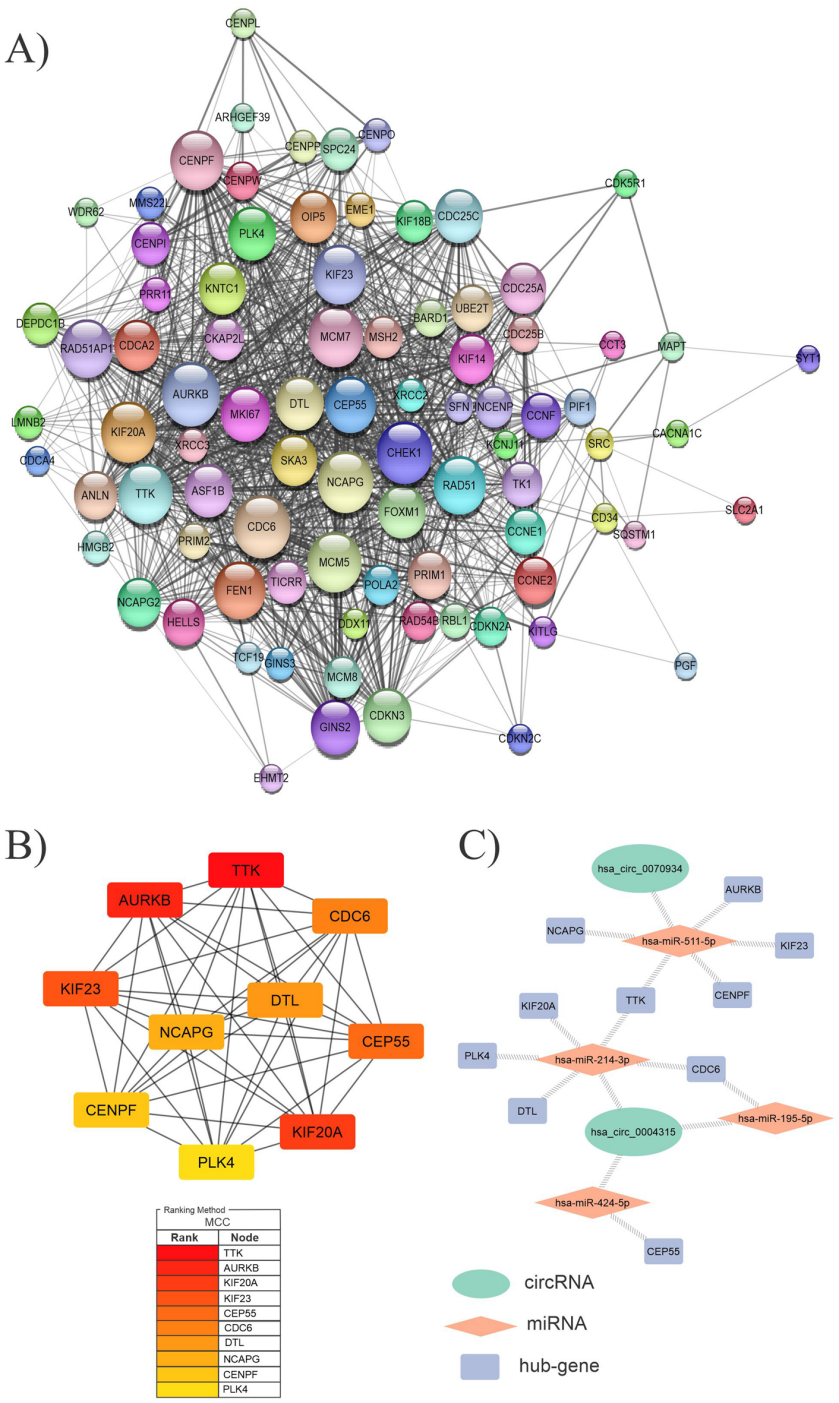
**(A)** A PPI network involving 204 genes has been associated with overall survival. The size of the node reflects the degree of value of the genes. (**B)** a network of ten hub genes. MCC scores were used to determine the top ten hub genes in the PPI network using the CytoHubba plugin of the Cytoscape software. The figure shows the significance of hubs on a color scale ranging from red to yellow, with red being the most important and yellow being the least important. (**C)** Network shows the interactions between selected DECs, FImiRNAs, and hub genes. DECs, FImiRNAs, hub genes are shown in green, red, and blue, respectively.

Using the STRING database, we constructed a PPI network to assess the biological interactions of 204 genes associated with the overall survival of the HCC patients. After removing unconnected nodes, the final PPI network had 86 nodes and 1133 edges. This network was visualized in Cytoscape (Fig. 9A), and the hub genes were detected using the cytoHubba plugin of Cytoscape. According to CytoHubba's MCC (Maximal Clique Centrality) ranking, the top hub genes were TTK, AURKB, KIF20A, KIF23, CEP55, CDC6, DTL, NCAPG, CENPF, and PLK4. (Fig. 9B) Following that, the DEC-FImiRNA-hub gene network was constructed, including two circRNAs (hsa_circ_0070934 and hsa_circ_0004315) and three miRNAs (hsa-miR-511-5p, hsa-miR-214-3p, and hsa-miR-424-5p) and hub genes (Fig. 9C).

## Discussion

Despite significant improvements in diagnosing hepatocellular cancer through various methods, appropriate novel strategies, such as identifying specific molecular regulatory mechanisms, are still required to optimize the treatments and diagnosis of HCC^33^. With the advent of the bioinformatics era and increased research on cancer-related genes, it has been demonstrated that there is a network of complex interactions between RNAs known as the ceRNA network (ceRNET) in the way that, focusing on this network and the factors involved, may promote the rapid diagnosis of various malignancies^34^.

Thousands of highly-conserved sequence circRNAs have been identified thanks to advances in high-throughput sequencing technology and computational biology techniques. These circRNAs, as potential regulatory elements, may act as ceRNAs in cell physiology, trapping miRNAs and thereby influencing the regulation of other transcripts^35^. Various studies have shown that circRNAs are expressed differentially in tumor tissues, including circRASGRF2^36^, CircPUM1^37^, and circGFRA1^38^, which have been related to HCC progression.

Similarly, there may currently be many circRNAs in HCC that need to be studied, identified, and assessed. CircRNAs, on the other hand, have excellent tolerance to exoribonuclease due to their covalently closed-loop structure. These molecules may be used as efficient diagnostic and therapeutic targets and promising biomarkers for prognosis^39^.

In this study, we first gathered three circRNA microarray datasets from the GEO database, and then we screened nine DECs in the initial stages. (Table 3) Other researchers have investigated the role of hsa_circ_0074854 and hsa_circ_0091570 in hepatocellular cancer^40,41^. However, the other seven DECs in liver cancer have yet to be explored.

**Table 3:** Comprehensive information of nine DECs.

circRNA	Alias	Gene symbol	Adj. p-value	p-value	LogFC	Regulation	Chrom
hsa_circRNA_103999	hsa_circ_0074854	MAT2B	2.99E−06	2.78E−08	1.91	Up	chr5
hsa_circRNA_104575	hsa_circ_0083766	EPHX2	1.23E−05	2.64E−07	1.54	Up	chr8
hsa_circRNA_001846	hsa_circ_0000520	RPPH1	1.90E−05	5.04E−07	1.78	Up	chr14
hsa_circRNA_104353	hsa_circ_0079958	HECW1	3.95E−05	1.37E−06	1.50	Up	chr7
hsa_circRNA_100477	hsa_circ_0016867	COG2	0.0002	1.07E−05	1.54	Up	chr1
hsa_circRNA_101740	hsa_circ_0008616	C16orf62	0.0002	1.31E−05	1.62	Up	chr16
hsa_circRNA_101873	hsa_circ_0004315	GLG1	0.0011	0.0002	1.53	Up	chr16
hsa_circRNA_103737	hsa_circ_0070934	LARP1B	0.0020	0.0005	1.78	Up	chr4
hsa_circRNA_105031	hsa_circ_0091570	MBNL3	0.0006	8.22E−05	−1.80	Down	ChrX

These seven DECs were abnormally upregulated in HCC cells based on our findings. According to the ceRNA hypothesis, increased expression of these seven DECs may decrease the expression of miRNAs inside the cell. We predicted DEC-related MREs using intersecting miRNAs from the CSCD database and downregulated DEMIs. The results revealed only four DECs (hsa_circ_0000520, hsa_circ_0004315, hsa_circ_0008616, hsa_circ_0070934) were ascertained as ceRNAs to downregulate miRNA expression inside the cell, and those DECs that did not have miRNAs matching with downregulated DEMI from TCGA were eliminated, such as hsa_circ_0083766, hsa_circ_0079958, and hsa_circ_0016867. The miRNAs affected by final DECs, including hsa-miR-542-5p, hsa-miR-326, hsa-miR-511-5p, hsa-miR-195-5p, hsa-miR-214-3p, and hsa-miR-424-5p, were referred to as FImiRNAs in this research.

Among these six FImiRNAs, miR-542-5p has a complementary sequence with MRE sites related to two DECs, hsa_circ_0000520 and hsa_circ_0008616. According to the previous research, miR-542-5p expression is downregulated in non-small cell lung cancer (NSCLC) tissues, which is implicated in NSCLC tumorigenesis^42^. This miRNA has also been investigated in breast cancer, endometrial carcinosarcoma, and osteosarcoma^43–45^.

For miR-326, some studies have found that this miRNA acts as a tumor suppressor in glioblastoma, human prostatic carcinoma, and breast cancer. The expression of miR-326 is downregulated in these cancers^46–48^. Besides that, this miRNA has been demonstrated to reverse chemoresistance in human lung cancer by targeting the specificity protein 1(SP1)^49^.

MiR-511-5p has been studied for its tumor-suppressive role in preventing colorectal cancer cell progression by targeting GPR116^50^ and lung adenoma-carcinoma cells by targeting oncogene TRIB2^51^. Also, a study has proven that miRNA-511-5p prevents malignant behaviors of breast cancer with a direct effect on SOX9 and PI3/AKT regulatory pathway^52^.

For miR-195-5p, overexpression of this miRNA has been demonstrated to inhibit cell migration and invasiveness in oral squamous cell cancer, cervical cancer, and breast cancer by targeting TRIM14, YAP1, and CCNE1 proteins, respectively^53–55^, or preventing thyroid carcinoma cell progression by acting on the p21/cyclin D1 axis^56^. Overall, miR-195-5p has the potential to be a tumor suppressor. MiR-214-3p and miR-424-5p, like the previously mentioned miRNAs, were investigated in some research for their involvement in reducing cell progression through their effect on transcripts and signaling pathways. MiR-214-3p, for example, serves as a tumor suppressor by targeting ABCB1 and XIAP proteins, preventing multi-drug resistance and stimulating apoptosis. MiR-424-5p as well suppresses intrahepatic cholangiocarcinoma metastasis by targeting ARK5^57–61^. In summary, the outcomes of this study on these miRNAs are similar to other studies, and these miRNAs may play a critical role in the development of HCC.

To determine the effects of DECs on mRNAs through miRNAs, an intersection was performed between the overlapped target genes of six FImiRNAs from the Mirwalk and Targetscan databases and upregulated DEGs from the TCGA database; as a result, 543 genes were obtained and named as FImRNAs. Afterward, a circRNA/miRNA/mRNA regulation network was established as a ceRNA network based on DEC‐FImiRNA and FImiRNA‐FImRNA interactions.

The GO and KEGG databases were employed to understand the biological roles and potential function of 543 FImRNAs. Based on GO analysis, these genes in the biological process are mainly enriched in the positive regulation of the cell cycle, DNA replication, and nuclear division. Therefore, they may play pivotal roles in tumorigenesis and tumor development. At the same time, KEGG analysis showed that these genes were mainly enriched in the ECM-receptor interaction, cell cycle, and Rap1 signaling pathway. Some studies have examined these pathways and shown many roles of these genes in cancer progression. In conclusion, the circRNAs explored in this study may perform similar or related functions via the circRNA-miRNA-mRNA axis.

Rap1 has a variety of functions in tumor initiation and development. Rap1 regulates integrins and cadherins by stimulating EGFR and Src/FAK, which plays essential roles in cell adhesion to ECM and cell–cell adhesion; both are crucial for tumor cell invasion and metastasis. Also, Rap1 induces tumors and epithelial-mesenchymal transition (EMT) via notch signaling. During tumorigenesis, the interaction between cancer cells and the tumor microenvironment (TME) causes ECM stiffness and alteration of ECM key receptors, leading to aberrant mechanotransduction and malignant transformation.

On the other hand, Src triggered by Rap1 signaling may activate the MAPK/ERK pathway, which has been found in some research to promote G0/G1 to S phase cell cycle progression and angiogenesis in cancer^62–65^. Thus, these FImRNAs' function and signaling pathways are associated with the occurrence and development of tumors. In summary, these FImRNAs, which are indirectly regulated by the four selected DECs we identified, may play a vital role in the HCC signaling pathway.

**Figure 10:**
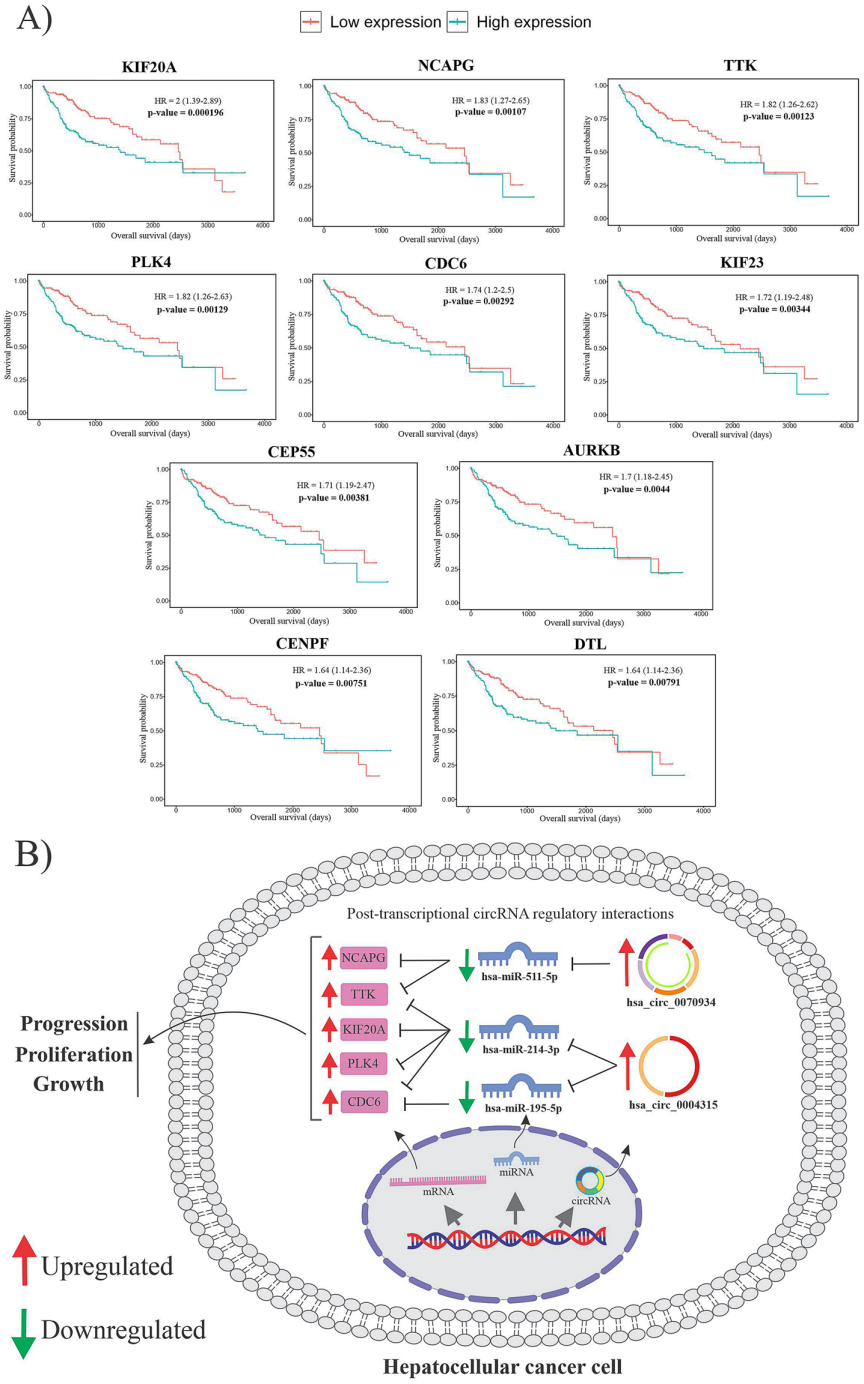
**(A)** The overall survival time of ten hub genes. In Kaplan–Meier plots, red lines indicate patients with lower hub gene expression, whereas green lines indicate greater hub gene expression. (**B)** The figure demonstrates a schematic illustration of the DECs-FImiRNAs-FImRNA regulatory network for five final hub genes located inside the cytoplasm. The upregulation of two DECs causes the downregulation of three miRNAs, limiting miRNA-mediated hub gene degradation, and HCC may develop due to hepatic overexpression of these five hub genes.

When 543 FImRNAs and 441 survival-related DEGs were intersected, only 204 FImRNAs were determined to be effective in survival. We built a PPI network for these 204 genes and selected ten hub genes from the network for further analysis. Figure 10A shows the overall survival time of ten hub genes in Kaplan–Meier plots. A circRNA-miRNA-mRNA regulatory axis was created for these hub genes to help the researchers understand them better. The top five hub genes based on the most significant p-value among these ten hub genes that considerably affect overall survival are KIF20A, NCAPG, TTK, PLK4, and CDC6.

KIF20A mRNA, which encodes protein MKlp2, has been reported to be significantly expressed in large human hepatocellular cells. MKlp2 accumulation is linked to abnormal hepatocyte proliferation and tumor aggressiveness in human hepatocellular carcinoma^66^. Interestingly, several studies showed a correlation between KIF20A overexpression and tumor progression and proliferation in colorectal cancer, renal clear cell carcinoma, and bladder cancer cells^66–68^.

NCAPG expression is increased in various cancers, including hepatocellular liver cancer, as an oncogene that stimulates cell proliferation and apoptosis through the PI3K/AKT/FOXO4 pathway. Another study found that knocking down NCAPG as a mitotic gene may prevent HCC cell growth, progression, and migration^69,70^.

According to the results of the previous studies, TTK activates Akt/mTOR, and MDM2/p53 in a p53-dependent mechanism increases cell proliferation and migration, which stimulates malignancy^71^. TTK overexpression has also been shown to increase HCC cell drug resistance to sorafenib, suggesting it may be a viable therapeutic target for human hepatocellular carcinoma^72^.

Concerning PLK4 and CDC6, their association with HCC has been described in previous researches; for example, PLK4 overexpression promotes cell growth, while its knockdown suppresses progression, invasion, and migration. CDC6 dysregulation has a role in developing many cancers, like hepatocellular carcinoma. CDC6, which acts as a regulator in the early stages of DNA replication and as a checkpoint controller before mitosis, has been associated with the clinical progression of HCC and may be utilized as a biomarker in patients with this kind of cancer^73,74^.

Finally, a circRNA-miRNA-mRNA regulatory axis based on DEC-FImiRNA and FImiRNA-hub gene interactions was built. This network, which contains two DECs (hsa_circ_0070934 and hsa_circ_0004315) and three FImiRNAs (hsa-miR-511-5p, hsa-miR-214-3p, and hsa-miR-195-5p), along with five final hub genes, may play an essential role in the development and progression of HCC (Fig. 10B).

Da-Wei Zhang and colleagues found that knocking down hsa_circ_0070934 expression inhibited CSCC cell proliferation and increased apoptosis compared to the negative control group. They also identified the importance of hsa_circ_0070934 in regulating HOXB7 expression levels by acting as a ceRNA and interacting with miR-1236-3p in the pathogenesis of CSCC. As a result, they discovered that hsa_circ_0070934 could be useful in the diagnosis and treatment of CSCC and other diseases^75^. For hsa_circ_0004315, some studies have found that this circRNA may act as a molecular sponge for miRNAs such as hsa-miR-214-3p and hsa-miR-195-5p to regulate the expression, respectively, of PPARGC1A and CCNE1 in hepatocellular carcinoma and breast cancer^76,77^.

Our results suggest that these two DECs identified by bioinformatic techniques may be utilized as effective diagnostic and valuable prognostic biomarkers.

## Conclusions

Our study was mainly conducted through bioinformatics analysis using software tools and genetic databases. To summarize, the goal of this study was to provide a comprehensive picture of the molecular mechanisms involved in HCC. Based on the ceRNA hypothesis, a circRNA/miRNA/hub gene network was constructed, and its function was predicted and evaluated. DECs, DEMIs, and DEGs inside this network may significantly affect patients' prognosis and survival. In conclusion, these genes, especially two final DECs include hsa_circ_0070934 and hsa_circ_0004315, may serve as novel prognostic factors in the HCC patients. These genes’ abnormal expression and function need to be verified in future laboratory studies (Supplementary Information).

## Supplementary Information

Supplementary Figures.

## Data Availability

The authors declare that the data supporting the findings of this study are addressed within the article.
