# Supplementary material for: Identification of potentially functional circular RNAs hsa_circ_0070934 and hsa_circ_0004315 as prognostic factors of hepatocellular carcinoma by integrated bioinformatics analysis
Source: Sci Rep. 2022 Mar 23;12:4933. doi: 10.1038/s41598-022-08867-w (PMC8943026; doi:10.1038/s41598-022-08867-w)
Supplement: Supplementary file 1 — Supplementary Figures. [file 41598_2022_8867_MOESM1_ESM.docx]

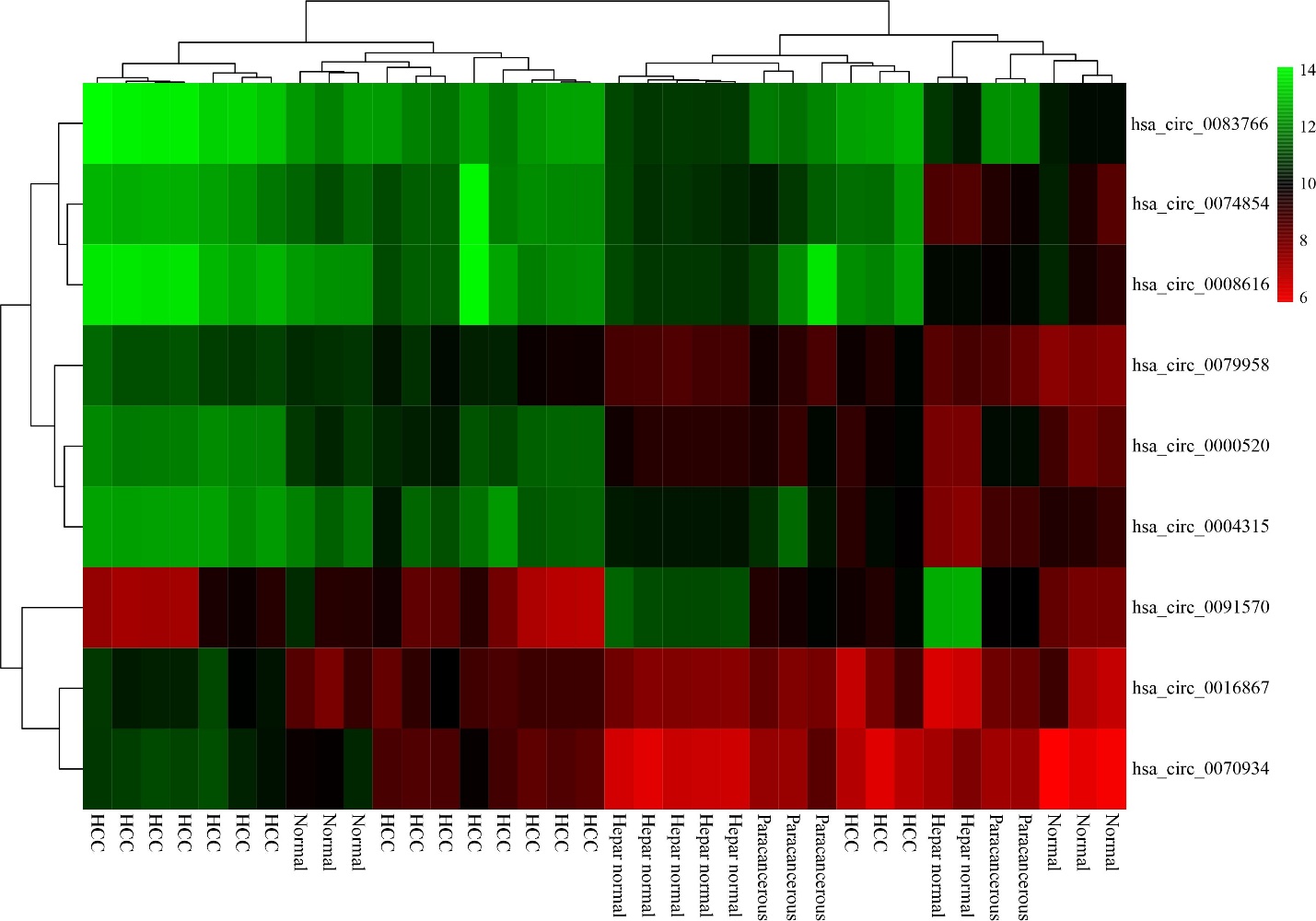


**Supplementary figure 1)** The heatmap shows the expression of nine circRNAs that were differentially expressed in HCC and normal tissues. The color change indicates the differences in the expression of the circRNAs.


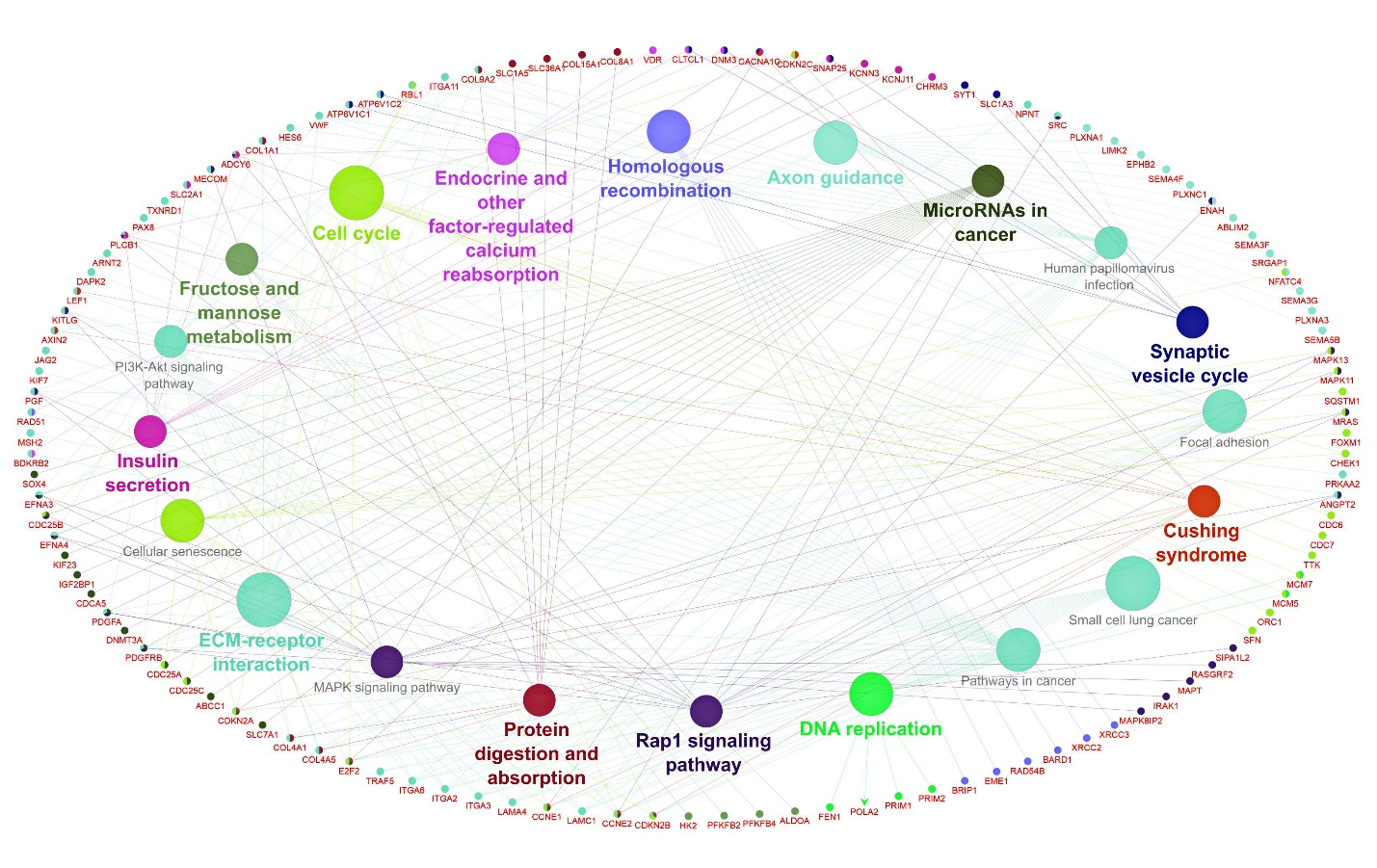


**Supplementary figure 2)** The ClueGO plugin in Cytoscape was used to identify enriched KEGG pathways, and a network of related KEGG terms was constructed. The connections of the pathways in the network are defined by functional nodes and edges shared by the FImRNAs, with a kappa score of 0.4. Only significant pathways (p-value ≤ 0.05) are shown in the enrichment network. The color code of the node shows the functional class in which it is engaged. The different colors indicate different molecular pathways involved in the enrichment analysis of the found FImRNAs. The bold terms represent the most critical functional pathways that determine the names of each group's signaling pathways. The names of the FImRNAs involved in each cluster are shown in the around with the red fonts.
